# “As du Coeur” study: a randomized controlled trial on physical activity maintenance in cardiovascular patients

**DOI:** 10.1186/s12872-018-0809-1

**Published:** 2018-05-02

**Authors:** Marion Fournier, Rémi Radel, Laurent Bailly, Christian Pradier, Roxane Fabre, Alain Fuch, Philippe Mossé, Jean-Jacques Domerego, Jocelyn Gal, Fabienne d’Arripe-Longueville

**Affiliations:** 1 Université Côte d’Azur, LAMHESS, Nice, France; 2 Department of Public Health, Centre Hospitalier Universitaire de Nice, Nice, France; 3 Régime Social des Indépendants, Nice, France; 4 Laboratoire LEST CNRS, University of Aix-Marseille, Marseille, France; 5 Hôpital Privé Gériatrique les Sources, Nice, France; 6 Centre Antoine Lacassagne, Nice, France; 7 EA Cobtek, University of Nice Sophia-Antipolis, Nice, France

**Keywords:** Habits, Physical activity, Cardiac rehabilitation, Randomized controlled trial

## Abstract

**Background:**

The benefits of supervised physical activity programs in cardiac rehabilitation have been amply demonstrated, but the quantity of physical activity often declines quickly once supervision ends. This trial assesses the effectiveness of an experimental intervention drawing on habit formation theory to maintain physical activity.

**Methods:**

Cardiovascular patients (*N* = 47) were randomly assigned to one of two groups. The first group participated in two supervised physical activity (SPA) sessions per week for 20 weeks. The second group was offered a progressively autonomous physical activity (PAPA) program as follows: the same supervised program as the SPA group for 10 weeks and then a further 10 weeks with one supervised session replaced by a strategy to build and sustain the habit of autonomous physical activity. The International Physical Activity Questionnaire (IPAQ; Craig et al. Med Sci Sports Exerc 35(8):1381–1395, 2003) was used to measure the quantity of physical activity, which was the primary outcome. The number of participants was limited, and we thus took multiple IPAQ measurements (at 0, 5, 7, 9 and 12 months after the start of the intervention) and used a mixed model for analysis. Physical condition, automaticity of the physical activity behavior, motivation, and quality of life were examined for changes.

**Results:**

No significant between-group differences were noted for physical activity behaviors after the program, physical condition, motivation, or behavioral automaticity. The PAPA group nevertheless completed more PA sessions during the intervention, and their quality of life was significantly higher than that of the SPA group at 12 months.

**Conclusion:**

Although the number of supervised sessions was lower, the progressively autonomous PA program resulted in the same or even higher positive outcomes than the fully supervised PA program.

**Trial registration:**

Current Controlled Trials ISRCTN77313697, retrospectively registered on 20 November 2015.

## Background

Cardiovascular disease (CVD) is the leading cause of death worldwide, accounting for 30% (16.7 million) of all deaths [50]. It has thus become crucial to find new ways of reducing its consequences. In order to encourage the development and maintenance of healthy behaviors, CVD patients are usually offered a cardiac rehabilitation (CR) program, which is a complex secondary prevention intervention. According to the guidelines for this type of intervention, physical activity (PA) should be a major component [2].

Although studies have shown that supervised PA programs for CVD patients improve the level of PA, they also indicate that the patients tend to stop their PA practices once the supervised period is over [45]. The post-CR literature, in fact, clearly points to the conclusion that PA behaviors decline rapidly soon after the CR ends (e.g., [13]). In a meta-analysis, Chase [9] reviewed 14 intervention programs to maintain or increase PA after CR. Although all the programs used behavioral or cognitive methods to enhance PA maintenance, none of them took habit formation into account, even though recent studies have identified it as the main predictor of maintaining health behaviors [17, 38]. To date, few interventional studies have concentrated on the habit framework in the health domain.

In order to form a habit, a behavior must be repeated many times, in the same context and over a long period [21]. It may even be more challenging to make PA behaviors habitual, given their complexity. Both Verplanken and Melkevik [48] and Phillips and Gardner [32] have nevertheless emphasized that the key step is simply making the decision to go to the exercise setting, an act that Phillips and Gardner [32] called exercise instigation. According to these authors, the habit strength of exercise instigation is an important predictor of exercise frequency. Nevertheless, no habit-based intervention has yet been tested to help individuals to maintain PA behaviors.

Habits have been shown to be context-dependent, which might explain why patients find it so difficult to continue their PA outside of the CR center. The program in the center not only provides supervision by trained coaches in a safe environment, but it also offers the possibility for social exchange. Once patients are back in their everyday home context, they are likely to miss this support and thus struggle to maintain PA behavior, with a frequent result being the return to sedentary habits. Numerous studies and reviews have compared the level of PA in center-based versus home-based interventions and shown no significant difference in the PA level when the program stops, whereas patients in long-term, home-based programs have shown better PA levels [11, 37, 43]. This suggests a great need for a program that fosters the transition between exercise in the CR center and the home. In this study of people with CVD, we hypothesized that a progressively autonomous PA (PAPA) intervention promoting habit formation in an everyday-life context would result in better PA maintenance than a fully supervised PA (SPA) intervention. We expected no between-group differences immediately after the end of the intervention, although we did expect that differences would emerge over time due to habit formation, with the PAPA group showing a slower and smaller decline in PA behaviors post-intervention than the SPA group. We also hypothesized that the habit-promoting PAPA program would yield better physical condition, motivation, automaticity of PA behavior, and quality of life at the end of follow-up compared with the SPA program.

## Method

The trial, registered as ISRCTN77313697, was a two-arm, randomized, open-label study in CVD patients to compare habit formation in a standard SPA-based CR program and the PAPA program.

### Sample size

We calculated the sample size with the simulation method of PASS 14 software (NCSS, Kaysville, UT, USA) for power analysis using a mixed model. An alpha risk of 5% and a bilateral hypothesis were taken into account for the power analysis. The expected scores of the participants in each arm of our study were estimated from the findings of large-scale studies [35, 37, 41] to assess post-CR PA behavior maintenance. These studies used a questionnaire that yielded an average score of PA minutes per week. As time has a linear effect, we assumed from the change in PA behaviors post-SPA that the weekly time spent in PA behavior would decline by 5 min every month following the intervention. This result was used to infer the PA level of the group at each post-program measurement point. We predicted no change in the amount of PA for the PAPA group, however, based on the findings of several studies showing that interventions designed to foster maintenance may stabilize or slightly increase PA behaviors over time (e.g., [1, 27, 35, 41]). The power analysis used a similar average score immediately after the intervention of 200 min/week for both groups, a general between-subject variability of 90 min/week, and an autocorrelation of the repeated measures of .55 [14]. After 1000 simulations, we concluded that 50 evaluable participants (25 participants in each arm) would be required to find the time x condition interaction effect with an 80% power level.

### Participants

#### Recruitment strategy

The study took place in the Alpes-Maritimes region of southern France; it started in 2014 and ran until January 2015 Fig. 1. We identified CVD patients from the electronic records of a public health insurance company for independent workers. They were contacted (*N* = 2248) by email and given with a full description of the study, including the research objectives, participant requirements, timetable, and testing and training facilities. If they were interested, they were asked to contact the insurance company. In October 2014, we were able to screen 91 volunteers by telephone, and they were given free complete medical checkups with the study cardiologist and underwent maximal effort testing and an electrocardiogram.

**Fig. 1:**
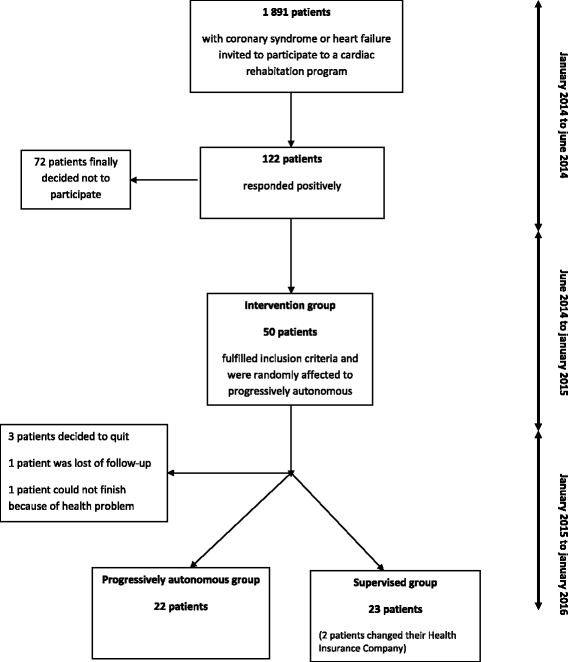
“As du cœur” study design

#### Inclusion criteria

Study participation was limited to adults over 18 years old who were registered in the health insurance database with chronic disease (CVD or cardiac insufficiency) and were deemed to have a low level of PA after brief physical activity assessment [24]. The other eligibility criteria were the agreement to attend two 60-min sessions per week at a fitness center and the cardiologist’s finding of no contraindication to PA. Forty-seven patients ultimately volunteered to enroll in the study.

### Consent

After a complete explanation of the study was provided in person, participants were asked to sign a consent form. They were encouraged to ask questions at this time and were assured of their right not to participate in the study or to withdraw from it at any moment without giving a reason.

### Randomization

The participants were randomized according to a 1:1 scheme using the CS randomization Clinsight software module (Ennov Clinical, San Francisco, CA, USA). A minimization method was used to prevent between-group imbalances for all known and unknown patient-related factors that might influence the study outcome. Patient age (< 65 years, ≥65 years), type of disease (coronary disease, cardiac insufficiency) and sex (male, female) were stratified.

### Blinding

Strict participant blinding is difficult in interventional PA studies, mostly for practical reasons related to the behavioral nature of the manipulation. We nevertheless used several strategies to limit contamination and prevent the biases associated with the lack of blinding [6]. We used Zelen’s [52] double-consent procedure adapted for behavioral intervention trials in patients with chronic disease [8]. Essentially, this meant that all eligible participants first signed a general consent form before receiving a second one that described all the terms associated with their type of treatment. In this way, we were able to mask the study details concerning the other arm from the participants. Also, to ensure blind evaluations, a separation was made between the staff in charge of outcome measurements and the staff delivering the intervention. Staff members who obtained outcome measurements were not informed of the group assignment. In addition, those responsible for data analysis were blind to group status.

### Ethics approval

Ethics approval for the trial was received from the National Ethics Committee for Human Research (ref: 14073) and the National Drug Agency (141299B-21). The regional governmental health agency also approved the various locations for the intervention (DOS-01115-0577-D).

### Intervention

The PAPA patients had two supervised sessions per week for the first 2.5 months and were advised to add at least one more session every week on their own to meet the American College of Sports Medicine (ACSM) guidelines for cardiac patients [26]. For the last 2.5 months, however, they had only one session per week and were encouraged to add two autonomous sessions every week. For the supervised sessions, the instructor was always the same and sessions were held at the same time of day and on the same day of the week. The sessions included Nordic walking (45 min to 1 h) at low to moderate intensity and circuit training (1 h) at moderate to high intensity. The intensity levels were set following the ACSM guidelines [26]. A heart rate monitor (Polar FT1 Heart Rate Monitor watch) measured each participant’s heart rate and the session contents were then individualized using these data: the resting heart rate and maximal heart rate were determined and training windows were calculated. In this way, we ensured that the participants in each group would be able to work at different intensities during the PA sessions. The instructor had been trained to use an autonomy-supportive coaching style (e.g., [44]) as autonomous motivation has been shown to strengthen habit formation of PA behaviors [16, 36]. Our strategies and tools were all chosen to support exercise instigation in terms of planning and preparatory behaviors [3], with the goal being to enhance autonomy, self-confidence, and knowledge and thereby facilitate home exercise. An individualized exercise prescription based on heart rate and current exercise capacity was therefore a core component of the intervention. Autonomous practice was promoted with support to aid habit formation. All received a pamphlet on PA (i.e., tips on safety rules, health benefits, nutrition, stretching, how to use a heart rate monitor, how to breathe) and a PA program containing a list of Nordic walking and cardio-training activities that could be done in their home environment. They also received the calendar of Gardner et al. [17]. This calendar is generic and can be used a tool for forming new habits. They were thus invited to use it to plan when and where they would do their PA sessions, with the expectation that they would write their session plans down. They were advised to put it where they could see it every day (e.g., on the fridge and/or a bathroom mirror) so that they would have frequent reminders. A researcher trained in habit formation theory then called them every 2 weeks for an approximately 15-min phone interview. The purpose was twofold: to obtain feedback about the autonomous PA sessions and to provide support for their efforts to build the PA habit. For example, the researcher helped the participants to choose an appropriate moment and an appropriate context for the autonomous PA sessions and reminded them to stick with their plans.

The SPA group had two supervised sessions per week over 5 months. They were also advised to carry out at least one more session on their own during the week to meet the American College of Sports Medicine (ACSM) guidelines for cardiac patients [26].

### Outcome measures

#### Demographics

Baseline demographic (sex, birthdate, zip code) and medical (CVD or cardiac insufficiency) data were collected.

#### Behavioral measures

The International Physical Activity Questionnaire (IPAQ) [10] has been extensively used in the literature to assess PA behavior. The participants thus responded to this self-report questionnaire at time 0, 5 months, 7 months, 9 months and 12 months after the start of the intervention. Their responses were then computed as energy requirements defined as METs (multiples of the resting metabolic rate). In addition, a subsample used a GT3X+ accelerometer for 1 week (score in METs) at T0, T5 and T12 to determine whether the self-reported PA measures correlated with the objective PA measures. The subsample comprised 30% of the participants selected randomly from among those who volunteered. It should be noted that a meta-analysis in other chronic diseases (e.g., [33, 34]) reported an acceptable level of agreement between the IPAQ and GT3X+, indicating a small to moderate association (*r* = .29). PA adherence was assessed by reports of their participation in the two supervised sessions per week and the autonomous sessions. A percentage was calculated for each participant.

#### Physical measures

General physical condition was measured at T0, T5 and T12 with the 6-min walking test (6MWT) [30], a handgrip strength test [40] and the sit to stand test [4].

#### Psychological measures

The psychological measures were made at 0, 5 and 12 months. We used the French adaptation of the Self-Report Behavioral Automaticity Index (SRBAI) [17], validated by Boiché [5], to assess the strength of the PA habits. The SRBAI is a four-item questionnaire (“I do it automatically”; “I do it without thinking”; “I do it without having to consciously remember”; and “I start doing it before I realize it”) that is typically used by calculating the average automaticity score. We assessed self-determined motivation with the sport motivation scale [7] to rate each participant’s motivation for PA behavior. [12]. Last, the SF-36 questionnaire [49] assessed any changes in quality of life, measuring life quality in terms of physical functioning, role-physical, bodily pain, general health, vitality, social functioning, role-emotional and mental health.

#### Statistics and data analysis

The statistical analyses were performed with a 5% alpha risk or 95% confidence interval using SPSS (IBM Statistics, Armonk, NY, USA). The normality of the quantitative data was evaluated using a graphical method (frequency histogram and quantile-quantile plot) and a Shapiro-Wilk test [18]. We tested the primary hypothesis on PA behavior maintenance using a mixed model procedure to take into account the non-independence of the repeated IPAQ measures grouped within participants. This is highly recommended for repeated measures analysis [42, 53]. The repeated measures were considered as a linear fixed factor (measurements at 5, 7, 9, and 12 months). The condition (PAPA vs. SPA) representing the criterion of the analysis was considered as another fixed factor in the model. The interactions between these two factors were examined to determine whether the change in PA behaviors after the end of the intervention differed with the arm of the trial. The initial level of PA (IPAQ at T0) was included as a fixed covariate to control for individual differences in PA levels. The intercept was defined as a random factor that could vary for each participant. For the secondary objectives, a general linear model (GLM) tested the hypotheses. The GLMs included the time of measurement (within-subject factor), the experimental condition (between-subject factor) and the interaction between these two factors. An independent-samples t-test was conducted to compare program adherence in the PAPA and SPA groups.

## Results

Mean ± standard deviation age for all patients was 63 ± 9.4 years (min = 42 years, max = 83 years) with 6.6% female patients (*n* = 3); 93% had coronary artery disease, 4.4% had cardiac insufficiency, and 2.2% had both. The SPA group had 23 participants (21 males, 2 females; 63.5 ± 8.1 years) and the PAPA group had 22 participants (21 males, 1 female; 62.5 ± 10.7 years). Five participants were lost to follow-up (2 for medical reasons, 3 for personal reasons). At the end of the 12-month follow-up period, 18 participants from the PAPA group and 19 from the SPA group completed the evaluation.

### Physical activity

**Fig. 2:**
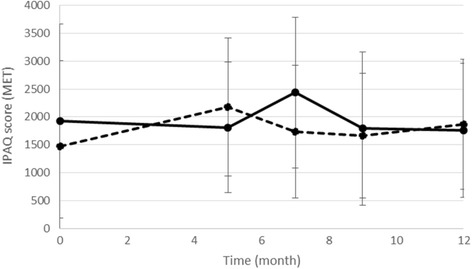
Changes in the self-reported physical activity (IPAQ score) over time for the progressively autonomous physical activity group (PAPA; solid line) and the supervised physical activity group (SPA; dashed line). The gray area represents the intervention period and the blank area represents the follow-up period. Error bars represent the standard deviation of the mean

The primary outcome was the level of PA according to the IPAQ questionnaire. We obtained 202 measures for the IPAQ across the five times of measurement. We kept only the measures greater than 0 and less than 5355 METS, as these values were identified as outliers [20]. One hundred and seventy-one measures were left for analysis after this screening. Because the data did not follow a normal distribution but presented strong right asymmetry (skewed toward higher values), a generalized linear mixed model (GLMM) was used as this model is an extension of the LMM for distributions that are not normal. We used a GLMM for gamma distribution with a log link as this model is adapted to positive values with right asymmetry. No condition effect was found [*F*(1, 106) = 0.446, *p* = .506], indicating that the groups had similar PA values. Whereas the model indicated a significant effect of time [*F*(1, 106) = 6.521, *p* = .012], suggesting a significant decline over time (coefficient = − 0.041), the absence of a time x condition effect indicated that this decline did not differ with the condition [*F*(1, 106) = 0.171, *p* = .680]. Figure 2 illustrates this pattern of results. Not enough complete data were collected (*N* = 5) for analysis of the objective measure of PA (accelerometer GT3X+), as participants reported trouble wearing the accelerometer for every waking hour over 7 days.

**Fig. 3:**
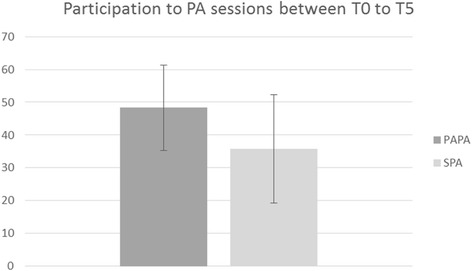
Participation in PA sessions for the PAPA and SPA groups between T0 and T5. Error bars represent the standard deviation of the means

**Fig. 4:**
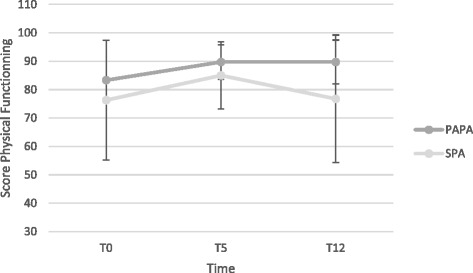
Changes in quality of life (score for physical functioning dimension) over time for the PAPA and SPA groups. Error bars represent the standard deviation of the means

### Participation in PA sessions

At the end of the intervention, the mean participation in the PA sessions (supervised or autonomous) at 5 months was 53.5%, and this was significantly different between the two groups (PAPA: 48.30 ± 13.07; SPA: 35.8 ± 16.52; t(43) = 2.83; d = 0.845; *p* = .007). In other words, the participants from the PAPA group completed significantly more PA sessions during the intervention than those in the SPA group (Fig. 3).

### Physical condition

Forty-four participants were evaluated for physical condition at 12 months (2 were excluded for health reasons and 1 for personal reasons). The results of the GLM (Table 1) indicated a time effect for heart rate suggesting that heart rate significantly decreased over time for both groups. However, the significant interaction of time and condition indicated that heart rate did not further decline after the intervention for the SPA group, whereas it continued to decline throughout follow-up for the PAPA group. A time effect was also found for the 6MWT, indicating that walking distance increased during the intervention but not during the follow-up. The PAPA group maintained their best walking distance during the follow-up period, but the SPA group did not. A time effect for the sit to stand test was also observed, increasing from 0 to 5 months for both groups. A condition effect for heart rate was found after the 6MWT, suggesting that the PAPA group generally had a higher heart rate after the 6MWT than the SPA group.

**Table 1:** Descriptive statistics (Panel A) and results of the statistical tests (Panel B) for physical and psychological measures at 0, 5 and 12 months for PAPA and SPA groups

Panel A				0 (Months)				5				12			
				PAPA		SPA		PAPA		SPA		PAPA		SPA	
				M	SD	M	SD	M	SD	M	SD	M	SD	M	SD
Heart rate				68.09	10.5	60.28	11.56	60.41	9.45	57.96	7.52	59.77	10.15	59.83	9.00
6-min walking				591.68	85.34	525.16	81.87	621.41	74.58	586.22	86.32	621.61	106.12	563.7	92.64
Cardiac frequency after 6 min. Walking				119.82	26.91	102.52	21.94	123.77	20.31	116.08	27.22	125.55	39.12	103.71	24.02
Sit-to-stand test				18.45	4.52	16.84	5.64	23.36	5.50	22.61	5.99	23.01	6.28	20.26	6.39
Habits (SRBAI)				17.00	10.28	16.68	11.67	22.89	9.26	26.45	11.03	22.88	10.03	22.55	11.65
Self-determination (SMS)				12.68	5.89	11.27	7.03	15.55	4.05	14.91	4.83	16.27	5.06	14.45	4.03
SF-36 (Physical functionning)				83,33	13,35	76,304	21,12	89,74	6,12	85,00	11,81	89,72	7,76	76,75	22,49
SF-36 (Role Physical)				66,67	33,85	51,087	35,74	63,16	38,52	71,25	30,65	70,83	37,62	61,25	41,73
SF-36 (Role-Emotional)				73,02	37,44	73,913	34,75	82,46	34,01	86,67	22,69	87,04	23,26	78,33	29,17
SF-36 (Vitality)				60,71	16,32	55,707	19,85	70,72	13,83	63,13	18,01	64,58	15,16	57,50	23,08
SF-36 (Mental Health)				68,81	17,10	67,826	13,97	73,95	18,38	73,75	14,50	75,28	16,13	70,25	17,36
SF-36 (Social functionning)				79,37	29,77	71,739	26,32	83,33	23,57	85,00	17,85	84,26	22,49	78,33	23,63
SF-36 (Bodily pain)				82,04	21,55	59,379	25,22	79,55	19,17	70,29	22,69	72,86	25,12	62,86	31,57
SF-36 (General Health)				58,75	16,20	54,087	21,21	68,95	16,61	66,35	19,59	65,11	13,31	58,40	22,02
Panel B	Time						Condition			Time×Condition					
	F	*p*-value	η^2^	Post-hocs (p-value)			F	p-value	η^2^	F	p-value	η^2^	Post-hoc (p-value)		
				0 vs. 5	0 vs. 12	5 vs. 12							SPA vs. PAPA at 0	SPA vs. PAPA at 5	SPA vs. PAPA at 12
Heart rate				.01	.06	.36							.072	.115	.469
6-min walking	14.73	.00	.49	.000	0.54	.09	2.43	.13	.07	2.10	.14	.12	.006	.483	.108
Cardiac frequency after 6 min. Walking	0.311	.73	.025	.45	.93	.52	7.35	.01	.21	1.65	.21	.06	.024	.115	.469
Sit-to-stand test	47.03	.00	.752	.000	.001	.738	.911	.347	.028	.755	.478	.046	.337	.553	.110
Habits (SRBAI)	5.117	.012	.237	.003	.024	.293	.021	.886	.001	1.33	.278	.075	.925	.284	.924
Self-determination (SMS)	3.206	.053	.16	.057	.016	.807	2.11	.155	.057	.494	.614	.028	.471	.658	.232
SF-36 (Physical functionning)	4.91	0.013	0.22	0.012	0.834	0.091	3.23	0.081	0.085	2.2	0.127	0.11	0.71	0.17	0.027
SF-36 (Role physical)	0.25	0.779	0.01	0.497	0.592	0.934	0.02	0.876	0.001	1.22	0.306	0.07	0.523	0.349	0.556
SF-36 (Role-Emotional)	1.44	0.252	0.08	0.137	0.145	0.837	0.06	0.814	0.002	0.82	0.451	0.05	0.966	0.658	0.268
SF-36 (Vitality)	6.37	0.005	0.27	0.002	0.224	0.013	1.63	0.211	0.044	0.03	0.971	0.002	0.325	0.185	0.283
SF-36 (Mental Health)	2.6	0.089	0.13	0.045	0.057	0.632	0.51	0.482	0.014	0.42	0.663	0.02	0.671	0.816	0.32
SF-36 (Social functionning)	0.6	0.552	0.03	0.275	0.412	0.802	0.53	0.469	0.015	1.51	0.234	0.08	0.432	0.708	0.221
SF-36 (Bodily pain)	0.89	0.42	0.05	0.932	0.209	0.262	4.67	0.038	0.118	3.03	0.062	0.15	0.003	0.33	0.19
SF-36 (General Health)	6.33	0.005	0.28	0.001	0.093	0.014	1.36	0.251	0.039	0.31	0.735	0.02	0.25	0.507	0.23

### Psychological measures

A time effect for self-determination and habit formation was observed, suggesting that they increased significantly for both groups between 0 to 5 months. The quality of life results of the SF-36 showed significant improvements at T5 in both groups on vitality (58.1 to 66.8, *p* = .005), general health (56.3 to 67.6, *p* = .005), and physical functioning (79.7 to 87.3, *p* = .013). At T12, physical functioning (*p* = .027) in the PAPA group only suggested that the intervention had a more positive impact on the life of the PAPA patients (Fig. 4).

## Discussion

The main findings of this study are as follows: The two groups showed no significant difference in physical activity behaviors after the program, nor did they differ in physical condition, motivation, or behavioral automaticity. However, the PAPA group completed more PA sessions during the intervention, and their quality of life was significantly higher than that of the SPA group at 12 months.

Our study confirmed that supervised CR is beneficial to patients, providing them with guidance in adopting safe PA practices [2]. However, supervised CR programs are sometimes found to be less effective than home-based programs at developing PA maintenance [11, 37, 43]. In addition, many patients cannot attend the supervised programs (20% of our sample) because they are costly and often involve considerable travel time. Yet home-based programs may not be adapted to those who lack confidence exercising on their own, those who have never had physically active lifestyles, and those with limited self-control [25]. We therefore see a need for programs that encompass aspects of both supervised and home-based programs and thereby provide a transition from the center to the home context. We hypothesized that a PA-based CR program starting with supervision in a center and then shifting to autonomous PA in the home would be even more beneficial for PA maintenance by ensuring the transfer of adequate PA practice to the everyday-life context. We therefore designed the PAPA intervention to help these patients form long-term PA habits that would more naturally carry over into the home environment.

In our comparison of the PAPA program and a supervised PA program, we expected to find a smaller decline in PA during the follow-up period for the participants of the PAPA group. However, we found no difference, as PA decreased slightly in both groups during the follow-up. The secondary objectives of this study were to determine whether the PAPA intervention led to better PA levels, physical condition, quality of life, strength of PA habits, and self-determined motivation for exercise during the follow-up period. We found no significant difference between the two groups for the strength of PA habits, self-determined motivation, and PA level. We observed a significant increase in physical condition over time for both groups, with a general increase after the PA intervention, but no difference between groups. Interestingly, the number of sessions performed during the intervention differed significantly, with more PA sessions completed by the PAPA group participants, suggesting that these patients were considerably more assiduous.

Significant differences were also found between the two groups on quality of life. Physical functioning (*p* = .027) was significantly improved in the PAPA group at 12 months in comparison to the SPA group. In other words, participants in the PAPA program had a better quality of life at the end of the follow-up. These results suggest that the PAPA participants, who had better participation during the program, continued to exercise during the follow-up.

The measure of habits (SRBAI) did not show significant results, although we suspect that the participants may have started to create habits. The accuracy of measuring habit strength by questionnaire has been questioned as individuals are unlikely to have access or awareness of automatic behavior [15, 19, 31]. The participants from the PAPA group may have scored better on quality of life because they had started to create PA habits, which could be explained by at least mechanisms. First, to encourage exercise repetition, the participants from the PAPA group received SMS cues every day before their PA session. Many repetitions are needed before a behavior becomes a habit [29], especially in the PA domain [22, 32]. This procedure has been shown to be efficient in creating PA habits in workplace environments, with those receiving an SMS before their PA sessions creating habits more quickly [14]. This systematic cue may have facilitated the initiation of the PAPA participants’ behavior. As highlighted by Tobias [46], the impact of the reminders decreases as habits develop, which would explain why the participants had a better quality of life after 12 months. The SMS cues may have prompted the repetition of PA behavior for the PAPA participants.

Second, the phone calls to instigate the PA behavior may have served as environmental cues, which are crucial to PA habit formation [32]. The literature has shown that an engagement that deliberatively initiates the desired behavior is needed to form a habit [51]. Correlational studies have shown that the best predictor of PA habits is the decision to go. This decision is called an instigation [32] and it fosters the repetition of the behavior. Habits are defined as “actions that are triggered automatically in response to contextual cues that have been associated with their performance” [28]. A cue can be another action (e.g., washing hands before lunch) or contextual (opening the fridge when going into the kitchen). After sufficient repetition, the action is activated upon exposure to these external cues. The experimenter who called the participants once every 2 weeks helped them to find facilitators for their PA sessions (i.e., meeting up with people to exercise with, using reminders, using the pamphlet with examples of sessions they could do on their own). He helped them to plan their PA sessions in advance and to anticipate and deal with problems (i.e., weather or organizational problems). For example, he suggested they use a calendar, similar to the one introduced by Gardner et al. [17] for taking medications, and plan their sessions in advance for the following week, taking into account other obligations. They were told to replace a supervised session with an autonomous session at home to avoid missing a session. This instruction was based on the finding that missing a repetition of the desired behavior increases the time needed for habit formation [21]. Some of the participants thus exercised on their own while others needed a social context and sought out family, friends or people from the program (one group continued to see each other for the autonomous sessions that replaced the supervised sessions). The experimenter also provided intrinsic reinforcement by satisfying psychological needs [39, 47], based on a study showing that intrinsic motivation has a positive impact on habit formation [16].

Several limitations of the study may have contributed to the lack of significant differences between the two groups. As shown by Maiorana and Ntoumanis [23], a major challenge is the physical activity questionnaire, which is employed in epidemiological research but is of limited usefulness for measuring PA at an individual level. The IPAQ may not have been suitable for the CVD patients in the context of our study, as suggested by the wide variability in the responses and the extreme values. In addition, a discrepancy was noted between the PA measures at pre-screening and the start of the study. To be eligible, participants had to be physically inactive according to their score at the Marshall’s questionnaire of PA [24], which was administered individually by phone. Yet, when the IPAQ was completed without supervision at the beginning of the intervention, the scores indicated that many participants could instead be considered physically active. It is possible that when answering the questionnaire on their own, the patients had difficulty in understanding the questions correctly or had difficulty in accurately reporting their PA level based on the 7 days preceding the measurement.

A second important limitation concerns the small size of our groups. Despite widespread communication and a relatively high number of volunteers (122), we did not manage to recruit the required sample size (47 instead of 50 participants). The statistical power might thus have been too low to adequately test the hypothesis that a progressively autonomous PA program would result in greater PA maintenance than a fully supervised program. Moreover, there were significant differences at baseline for the 6MWT and heart rate after the 6MWT. This is unfortunate as group assignment had been randomized to avoid imbalances between the two groups. Therefore, we strongly encourage future studies to test this hypothesis using larger groups of participants and another measure of PA.

## Conclusion

In the last few years, increasingly more research has focused on the formation of such healthy habits as healthy eating, regular tooth brushing and not smoking. Few studies, however, have focused on PA [31]. To our knowledge, longitudinal studies on the features of habits have been sparse [51], especially in the PA domain. In addition, no interventional study has been conducted to examine habit formation in CVD patients. Our intervention study thus enriches the literature on habit formation. Although this study showed no significant difference between the PAPA and SPA groups in terms of post-program PA behaviors, physical condition, self-determined motivation, or behavioral automaticity, it clearly demonstrated that a progressively autonomous PA program yielded better participation in PA sessions at 5 months and better quality of life at 12 months follow-up, suggesting that the intervention on habit formation had an impact. Future work with a larger sample might confirm these promising results.

## Abbreviations

CR: Cardiac rehabilitation
CVD: Cardiovascular disease
PA: Physical activity
PAPA: Progressively autonomous physical activity
SPA: Supervised physical activity
